# 

**Published:** 1905-01

**Authors:** 


### CONTENTS.

ORIGINAL COMMUNICATIONS—

TUBERCULOSIS AS A SOCIOLOGICAL FACTOR. By R. R. Kime, M.D., Atlanta, Ga. .......... 649

CEREBRASTHENIA THE BANE OF THE AMERICAN BRAIN-WORKER. By E. Van Goidtsnoven, A.M., M.D., Atlanta, Ga .......... 658

PRESIDENT'S ANNUAL MESSAGE. By A. W. Stirling, M D., C.M. (Edin); D.P.H. (Lond.), Atlanta, Ga. .......... 664

EDITORIAL NOTES AND COMMENTS—

IS RADIOTHERAPY A FAD? .......... 669

THE STANDARDIZATION OF MEDICAL EXAMINING BOARDS .......... 671

NEWS AND NOTES .......... 673

CONTENTS CONTINUED .......... X

### *CONTENTS—CONTINUED.*

SELECTIONS AND ABSTRACTS—

The Nature and Treatment of Hepatic, Cardiac and Renal Dropsy............ 677
Some Observations on Hemoptysis.................................... 687
Inoculating the Ground.............................................. 693
New York County Medical Association................................. 696
Fecal Impaction..................................................... 701
The Function of the Cecum and Appendix.............................. 703
Management of Pruritus Ani.......................................... 705
The Correction of Abnormal Conditions of the Blood Relative to Surgical Operations.......................................................... 708
The Borderland of Consumption....................................... 711
Why I Give Preference to Buffalo Lithia Over all Other Mineral Waters...... 713
How Breakage of Bottles can be Reduced to a Minimum.................. 717
A Practical Suggestion for the Prevention of Ankylostomiasis......... 719

MEDICAL ITEMS—CONTINUED.........................xvi, xviii, xx, xxii, xxiv

